# Cortex-Wide Dynamics of Intrinsic Electrical Activities: Propagating Waves and Their Interactions

**DOI:** 10.1523/JNEUROSCI.0623-20.2021

**Published:** 2021-04-21

**Authors:** Yuqi Liang, Chenchen Song, Mianxin Liu, Pulin Gong, Changsong Zhou, Thomas Knöpfel

**Affiliations:** ^1^Department of Physics, Centre for Nonlinear Studies and Beijing-Hong Kong-Singapore Joint Centre for Nonlinear and Complex Systems (Hong Kong), Institute of Computational and Theoretical Studies, Hong Kong Baptist University, Kowloon Tong, Kowloon, Hong Kong, People's Republic of China; ^2^The HKBU Institute of Research and Continuing Education, Shenzhen 518000, People's Republic of China; ^3^Laboratory for Neuronal Circuit Dynamics, Imperial College London, London SW7 2AZ, United Kingdom; ^4^School of Physics, University of Sydney, Sydney 2006, New South Wales, Australia; ^5^Australian Research Council Centre of Excellence for Integrative Brain Function, University of Sydney, Sydney 2001, New South Wales, Australia; ^6^Department of Physics, Zhejiang University, Hangzhou 310027, People's Republic of China; ^7^Beijing Computational Science Research Center, Beijing 100193, People's Republic of China; ^8^School of Biomedical Engineering, Shanghai Tech University, Shanghai 201210, People's Republic of China

**Keywords:** cortex, dynamics, fluorescence, GEVI, imaging, mouse

## Abstract

Cortical circuits generate patterned activities that reflect intrinsic brain dynamics that lay the foundation for any, including stimuli-evoked, cognition and behavior. However, the spatiotemporal organization properties and principles of this intrinsic activity have only been partially elucidated because of previous poor resolution of experimental data and limited analysis methods. Here we investigated continuous wave patterns in the 0.5–4 Hz (delta band) frequency range on data from high-spatiotemporal resolution optical voltage imaging of the upper cortical layers in anesthetized mice. Waves of population activities propagate in heterogeneous directions to coordinate neuronal activities between different brain regions. The complex wave patterns show characteristics of both stereotypy and variety. The location and type of wave patterns determine the dynamical evolution when different waves interact with each other. Local wave patterns of source, sink, or saddle emerge at preferred spatial locations. Specifically, “source” patterns are predominantly found in cortical regions with low multimodal hierarchy such as the primary somatosensory cortex. Our findings reveal principles that govern the spatiotemporal dynamics of spontaneous cortical activities and associate them with the structural architecture across the cortex.

**SIGNIFICANCE STATEMENT** Intrinsic brain activities, as opposed to external stimulus-evoked responses, have increasingly gained attention, but it remains unclear how these intrinsic activities are spatiotemporally organized at the cortex-wide scale. By taking advantage of the high spatiotemporal resolution of optical voltage imaging, we identified five wave pattern types, and revealed the organization properties of different wave patterns and the dynamical mechanisms when they interact with each other. Moreover, we found a relationship between the emergence probability of local wave patterns and the multimodal structure hierarchy across cortical areas. Our findings reveal the principles of spatiotemporal wave dynamics of spontaneous activities and associate them with the underlying hierarchical architecture across the cortex.

## Introduction

During anesthesia and sleep but also in quiet wakefulness, the brain exhibits spontaneous activities that reflect self-organized intrinsic brain dynamics, which are independent from external stimuli application or task performance and, at least in the case of general anesthesia, do not relate to motor behavior. In recent decades, spontaneous activity has received increasing attention and has been described in various species (Xiao et al., 2017; Smith et al., 2018). Spontaneous activities can influence evoked responses (Azouz and Gray, 1999; Davis et al., 2020); contribute to observable variability in cognition and behavior (Romano et al., 2015); and play important roles in the consolidation of new memories, motor learning (Marshall et al., 2006), and sleep-dependent enhancement in visuomotor performance (Landsness et al., 2009). Studying the essential organization properties of spontaneous activity is thus vital for understanding brain functions (Deco et al., 2014; Capone et al., 2019; Chen and Gong, 2019).

The traditional way of characterizing spontaneous activity is based on correlation analysis. For instance, based on cross-correlations between a predefined seed region and other brain regions, whole-brain spontaneous activity exhibits spatially structured, patchy correlation patterns (Fox et al., 2005). However, increasing evidence has shown that spontaneous brain activity exhibits far richer spatial and temporal dynamics. Particularly, spontaneous activity is organized as propagating waves, which have been found at different neural levels by using different recording techniques, including multielectrode arrays, voltage-sensitive dye (VSD) imaging, electroencephalography (EEG), electrocorticography, magnetoencephalography, and functional magnetic resonance imaging (Freeman and Barrie, 2000; Garaschuk et al., 2000; Lee et al., 2005; Rubino et al., 2006; Wu et al., 2008; Huang et al., 2010; Patten et al., 2012; Alexander et al., 2013; Zanos et al., 2015). Several studies have described the origins, pathways, and recruitment of various cortical areas during the propagation of waves of activities (Nir et al., 2011; Shimaoka et al., 2017). However, recording techniques such as intracellular recordings and extracellular multiple-unit recordings have limited spatial coverage, and EEG has limited spatial resolution, hence the recruitment of neuronal populations during wave patterns has been evaluated mainly on the basis of the timing of local oscillation peaks. Thus, detailed spatiotemporal organization properties of spontaneous activity and the underlying mechanisms remain unexplored.

Optical voltage imaging data using genetically encoded voltage indicators (GEVIs) offers a high-spatiotemporal resolution readout of population membrane voltage in superficial layers across a large portion of the cerebral cortex of living mice (Knöpfel, 2012). This approach hence provides an opportunity to investigate the large-scale neural dynamics at sufficient spatiotemporal resolution and coverage. Recent studies using a similar VSD imaging method typically focus on specific sensory-evoked and corresponding spontaneous activity motifs (Mohajerani et al., 2013) or the existence of wave propagation (Muller et al., 2014). However, details of how waves are spatiotemporally organized are still missing. Using GEVI imaging of spontaneous voltage activity from pyramidal neurons in mice, here we addressed the following questions. (1) Are there any regularities in the seemingly random occurrence of complex spatiotemporal wave patterns at different spatial scales? (2) Can different types of waves coexist, and how do these coincident waves interact? (3) How does the formation of cortex-wide wave patterns relate to the underlying brain structure?

We address these questions on delta waves (0.5–4 Hz) with an advanced wave analysis method, which extracts both the spatial and temporal features of continuous wave patterns (Townsend et al., 2015). Although propagating patterns show a large variation between individual events, principal component analysis revealed that the top five principal modes already contribute to >80% of the variance. We found that the emergence of sources and sinks can reverse the propagating direction of large-scale traveling waves, and their interactions can generate saddle wave patterns. Moreover, sources, sinks, and saddles are preferentially formed at specific locations, intricately related to the cortical connectivity. These findings underpin the idea that spontaneous activity plays an important role in intracerebral communications and reflect details of the underling neuronal circuit mechanism.

## Materials and Methods

### 

#### 

##### Experimental design and statistical analyses.

We used mesoscopic transcranial voltage imaging datasets for the analysis. Data acquisition was as described previously (Akemann et al., 2010, 2012; Scott et al., 2014; Song et al., 2018). Briefly, CaMK2A-tTA;tetO-chiVSFP transgenic animals expressed the GEVI chimeric VSFP Butterfly (Mishina et al., 2014; Song et al., 2018) in pyramidal neurons across all cortical layers. The epifluorescence imaging approach we used here restricts optical access and signal detection to the superficial cortical layers (layer 2/3). Under surgical anesthesia, animals were implanted with a transcranial cortical window through a thinned but otherwise fully intact skull and a head-fixation plate. Image acquisition was performed using a dual-emission wide-field epifluorescence macroscope equipped with two synchronized CMOS (complementary metal oxide semiconductor) cameras, using high-power halogen lamps for fluorescence excitation (Moritex/BrainVision) and the following optics (Semrock): mCitrine (donor) excitation 500/24; mCitrine emission FF01-542/27; mKate2 emission BLP01-594R-25; excitation beam splitter 515LP; and detection beam splitter 580LP. The ratio of changes of gain-equalized (Akemann et al., 2012) fluorescence intensities acquired with the two cameras reflects the spatiotemporal dynamics of spontaneous membrane voltage fluctuations of populations of pyramidal neurons.

Datasets were acquired at a 150 Hz acquisition frame rate and over a cortex-wide two-hemisphere field of view. The original spatial resolution of the signal we extracted from the camera is 29 × 29 μm. During the imaging session (several trials of 180 s duration), mice were under anesthesia/sedation (induced by a bolus injection of 30 mg/kg pentobarbital sodium). With this protocol, animals are lightly anesthetized initially, and then recover over a state of sedation to wakefulness. This progression was monitored using the heart rate as a proxy. A brain state characterized by an absolute lack of spontaneous limb and whisker movements is referred to as “anesthesia.” We analyzed 13 experimental trials over five mice (mice 1–4 are males and mouse 5 is female), and each mouse provided several trials under the anesthesia condition (mouse 1, three trials; mouse 2, two trials; mouse 3, three trials; mouse 4, three trials; mouse 5, two trials. Different mice had different number of trials satisfying the condition of anesthesia). Each trial recorded spontaneous voltage activity for 180 s continuously.

MATLAB is used for data processing and analysis. Data and codes used to create all the plots are available on request.

##### Data preprocessing.

We extracted voltage signal from raw fluorescence signals as previously described (Akemann et al., 2012; Shimaoka et al., 2017; Song et al., 2018). Reliable computation of the phase velocity fields (PVFs) was achieved after reduction of spatial noise by 2 times coarse graining using bicubic interpolation (weighted average of pixels in the nearest 4-by-4 neighborhood; function *imresize*, scale = 0.5, MATLAB, MathWorks). The final spatial data size for the imaged field of view was a 44 × 52 matrix. The analysis method based on the phase wave requires that signals across multiple neighboring pixels occur with similar spectral properties. Thus, we focused on narrowband delta oscillation 0.5–4 Hz by bandpass temporal filtering (Chebyshev type II, function *filtfilt*, MATLAB, MathWorks) to improve the signal-to-noise ratio according to the signal power of the wavelet transform. Periods with large temporal fluctuations (amplitude, >3 SDs) of the filtered voltage signals were identified as movement artifacts and were excluded from further analysis. The phase velocity fields were calculated on the spatially and temporally filtered data.

##### PVF.

We characterized the cortex-wide spatiotemporal patterns using phase velocity field analysis, which was adapted from physical theories of turbulence and validated for data recorded with different techniques and modalities (Townsend et al., 2015; Townsend and Gong, 2018). Briefly, the method assumes that contours (isolines) of the phase of brain waves propagate monotonically spatiotemporally. First, we used Hilbert transform on the 0.5-4Hz bandpass-filtered voltage signal to extract instantaneous phase on each pixel$\phi \left(x,y,t\right)$. Phase velocity fields $v_{\varphi }\left(x,y,t\right)=(u\left(x,y,t\right),v\left(x,y,t\right))$ can be calculated by adapting the optical flow estimation method, which is based on the changes of phases between two consecutive frames, with phase constancy and spatial smoothness constraints. Both constraints are expressed together as a single minimization problem, which is described as the Euler–Lagrange equations (Bruhn et al., 2005). The toolbox NeuroPatt implementing the above method can be found on the website https://github.com/rorygt/NeuroPattToolbox.

To further validate our PVF method, we shuffled data by randomizing raw voltage signal temporally (keeping power spectrum on each pixel but randomizing the phase of the Fourier components) and spatially (randomly shuffling the pixels). Then we applied the same data-processing procedure to the shuffled data as for the real data.

##### Gradients of PVF.

Spatiotemporal patterns of neural activities should be distinguishable from patterns obtained with random shuffled data in terms of continuous spatial gradients in the PVF. We measured the gradients $\nabla v_{\varphi }(x,y,t)=\left(\frac{\partial v_{\varphi }(x,y,t)}{\partial x},\frac{\partial v_{\varphi }(x,y,t)}{\partial y}\right)$ at each pixel (Chebyshev type II, function *gradient*, MATLAB, MathWorks). The partial differentiation is obtained as the difference of the *x*-projection (or *y*-projection) of the velocity vectors at two neighboring pixels. Small gradients indicate high coherence in the speed and direction, as expected for an organized (nonrandom) activity pattern. At each time *t*, gradient of PVF was defined as spatial average of pixel-wise gradients, as follows:

$$|\bar{\nabla v_{\varphi }}|\left(t\right)=\frac{1}{N}\sum_{x,y}\left|\nabla v_{\varphi }(x,y,t)\right|,$$

where N is the number of pixels in the analysis window.

##### Identification of wave patterns.

We followed the method of identifying wave patterns as previously described (Townsend et al., 2015) with some modifications to detect different types of wave patterns, including plane wave, and source and sink patterns. We detected plane waves by using the order parameter of the PVF in the analysis window, as follows:

$$\bar{v_{\varphi }}\left(t\right)=\frac{1}{Nv_{0}(t)}|\sum_{x,y}v_{\varphi }(x,y,t)|,$$

where N is the number of pixels in the analysis window, $v_{0}$ is the average magnitude of the velocity over all pixels, and $v_{\varphi }$ is the phase velocity $v_{\varphi }(x,y,t)$. The order parameter $\bar{v_{\varphi }}$ ranges from 0 to 1, with 1 representing the case where the velocity vectors are parallel. We set a threshold $\bar{v_{\varphi }}\left(t\right)\geq$ 0.85 to identify plane waves in the examined areas. Variation of the threshold value between 0.8 and 0.9 did not substantially change the results. Relaxing the threshold to smaller values allowed us to capture largely coherent propagating waves within the analysis window that can coexist with regions of heterogeneous propagation directions corresponding to complex local wave patterns such as sources, sinks, and saddles (see below).

We defined standing waves (synchrony) as periods where there is no apparent wave velocity (i.e., propagation) across the analysis window. The criterion for standing waves was an average magnitude of the velocity fields 2 SDs below the mean value across the analyzed time period.

We first asked whether the PVF across the analysis window is stationary (standing wave), and only if not, applied the criteria to detect plane and complex waves. Plane waves are detected when the wave propagation directions are narrowly distributed within the analysis window. We examined how the emergence of plane waves depends on the size of the analysis window (see Fig. 5). In this context, we note that in a very small analysis window most nonstationary wave pattern would be detected as a plane wave. Our smallest analysis window has 16 regions of interest, and the largest probability of plane wave is 81% among five mice; hence, even with our smallest window plane waves are not detected in all PVFs. We also like to note that if waves were all (dominantly) large-scale waves, their detection probability would not increase (or increase a little) with decreasing window size.

Local complex wave patterns are organized around the critical points, which were identified by the intersections of two bilinearly interpolated null clines of the phase velocity field. Eigenvalues of the Jacobian matrix at the corners of the four pixels around the critical point were then used to further classify the pattern types into source, sink, or saddle, as follows:

$$J=\left(\begin{array}{cc} \frac{\partial u}{\partial x} & \frac{\partial u}{\partial y}\\ \frac{\partial v}{\partial x} & \frac{\partial v}{\partial y} \end{array}\right).$$

Based on the trace (τ) and determinant (Δ) of the Jacobian matrix, source (unstable point, τ > 0), sink (stable point,τ < 0) and saddle (Δ < 0) patterns were determined. If both eigenvalues are real and of the same sign, the hyperbolic equilibrium is a “node.” When eigenvalues are complex-conjugate, the hyperbolic equilibrium is a “focus.” Different from previous definitions (Townsend et al., 2015), we counted each unstable node or focus as a source event, and stable node or focus as a sink event. We used the singularity point of PVF with near-zero velocity to define the locations of a source or sink. The patterns were detected in the analysis window by the NeuroPatt toolbox (https://github.com/rorygt/NeuroPattToolbox).

To validate our method to detect sources, sinks, and saddles (local waves), we compared the probability of local waves detected in real data and shuffled data as a function of a detection threshold. This detection threshold was defined as a pair of values (*d, r*), where *d* is the duration (number of time steps) of the lifetime of the same local wave pattern and *r* is the minimum radius (number of pixels from the singularity) of the local wave pattern. Probability at detection threshold (*d, r*) is defined as the number of local wave patterns with detection threshold (*d, r*), divided by the number of local wave patterns with detection threshold (1, 1).

##### Singular value decomposition of wave patterns.

We applied singular value decomposition (SVD) to all phase velocity fields $v_{\varphi }\left(x,y,t\right)=(u\left(x,y,t\right),v\left(x,y,t\right))$ to identify the principal components of the wave patterns (Townsend and Gong, 2018). We first reorganized all phase velocity fields into a standard form and combined them into matrix w. At every time step, we had a 2D matrix ***A*** of PVF vectors [44 × 52]. In step 1, we extracted the real part of the matrix ***A*** and reshaped them into a 1D matrix ***B*** [1 × 2288]. We also extracted the imaginary part of the matrix ***A*** and reshaped them into a 1D matrix ***C*** [1 × 2288]. In step 2, we concatenated ***C*** to the end of ***B*** to get matrix ***D*** [1 × 4576]. In step 3, we repeated step 1 and step 2 on every time step and concatenated all the matrices ***D*** for the time length *L* to organize matrix w [*L* × 4576]. *L* equals the time steps of one 3 min data trial. The singular value decomposition can be defined as follows:

$$w=T\sum R^{*},$$

where T and R are unitary matrices, * denotes the conjugate transpose, and **∑** is a rectangular diagonal matrix of singular valuesσ. The kth spatial mode, defined by the velocity field in the kth column of R, has a proportion of the overall variance given by $\sigma_{k}^{2}/\sum_{i}\sigma_{i}^{2}$. The top three spatial modes with the largest variance accounted for 70.4% of the total variance in the data of one representative mouse shown in Results section. Across the data from the five mice analyzed the three spatial modes with the largest variance accounted for 44.5 ± 17.9% of the total variance.

Then we projected the instantaneous representative phase velocity fields on the principal modes, as follows:

$$M=\frac{w}{R},$$

where M is the weight matrix of every principal mode contributed to all phase velocity fields. From this, we can obtain the projection variance of the mth spatial mode on thenth phase velocity fields, as follows:

$$M_{m,n}^{2}/\sum_{i}M_{i,n}^{2}.$$

##### Probability of local wave patterns.

To investigate the spatial distribution of the emergence of local wave pattern types (source, sink, and saddle), we calculated the center location of every source, sink, and saddle pattern and the wave duration (the number of time steps that the same location is occupied by the same singularity). For each pixel and wave pattern type, we calculated the probability of pattern emergence as the cumulative time the pattern existed. After obtaining the probability of pattern emergence, we averaged across pixels for each cortical region as a measure of the experimental probability of pattern emergence of that region.

##### Functional registration of data into Allen Mouse Brain Atlas.

To study the relationship between wave patterns and the anatomic properties of the underlying circuitries (see Fig. 9), we used the Allen Institute Mouse Brain Atlas (www.brain-map.org) projected to our plane of imaging. The two cortical hemispheres were registered into the atlas by aligning the visual and whisker stimuli response centers in voltage imaging data (Song et al., 2018) to the center of primary visual area and primary somatosensory area-barrel field of the atlas correspondingly (function *imregtform, imwarp*, MATLAB, MathWorks).

##### Correlation of complex wave patterns to the mouse cortical architecture.

Next, we examined how the occurrence probability of the complex wave patterns relates to the underlying cortical architecture. We first calculated the singularity probability for each wave pattern type and for each pixel. Then, we averaged across cortical region, pooling across the two hemispheres to generate a regional probability map. To relate these regional probabilities to the underlying cortical architecture, we used the hierarchical gradients of the mouse cortex (Fulcher et al., 2019) as a structural estimate. We calculated the Pearson's linear correlation coefficient between the average (*N* = 13 trials; five mice) probability of each wave type against the hierarchical gradient index of the corresponding cortical region (a total 21 cortical regions common to both data types). To investigate whether this correlation was by chance, we randomly shuffled the locations of singularity probabilities of each pixel and of each pattern type, averaged across cortical regions and pooled across the two hemispheres to generate randomly shuffled regional probability maps. For each randomly shuffled probability map, we calculated the Pearson's linear correlation coefficient with the hierarchical gradient index. The correlation coefficient calculated with the actual data lies clearly outside the distribution of the correlation coefficients obtained from 1000 shuffled datasets (see Fig. 9*E*). This analysis strongly suggests that the correlation between localizations of small-scale activity waves and cortical architecture, as reflected in the hierarchical gradient index, is not by chance.

## Results

### Complex spatiotemporal wave patterns revealed by phase velocity field analysis

**Figure 1.**
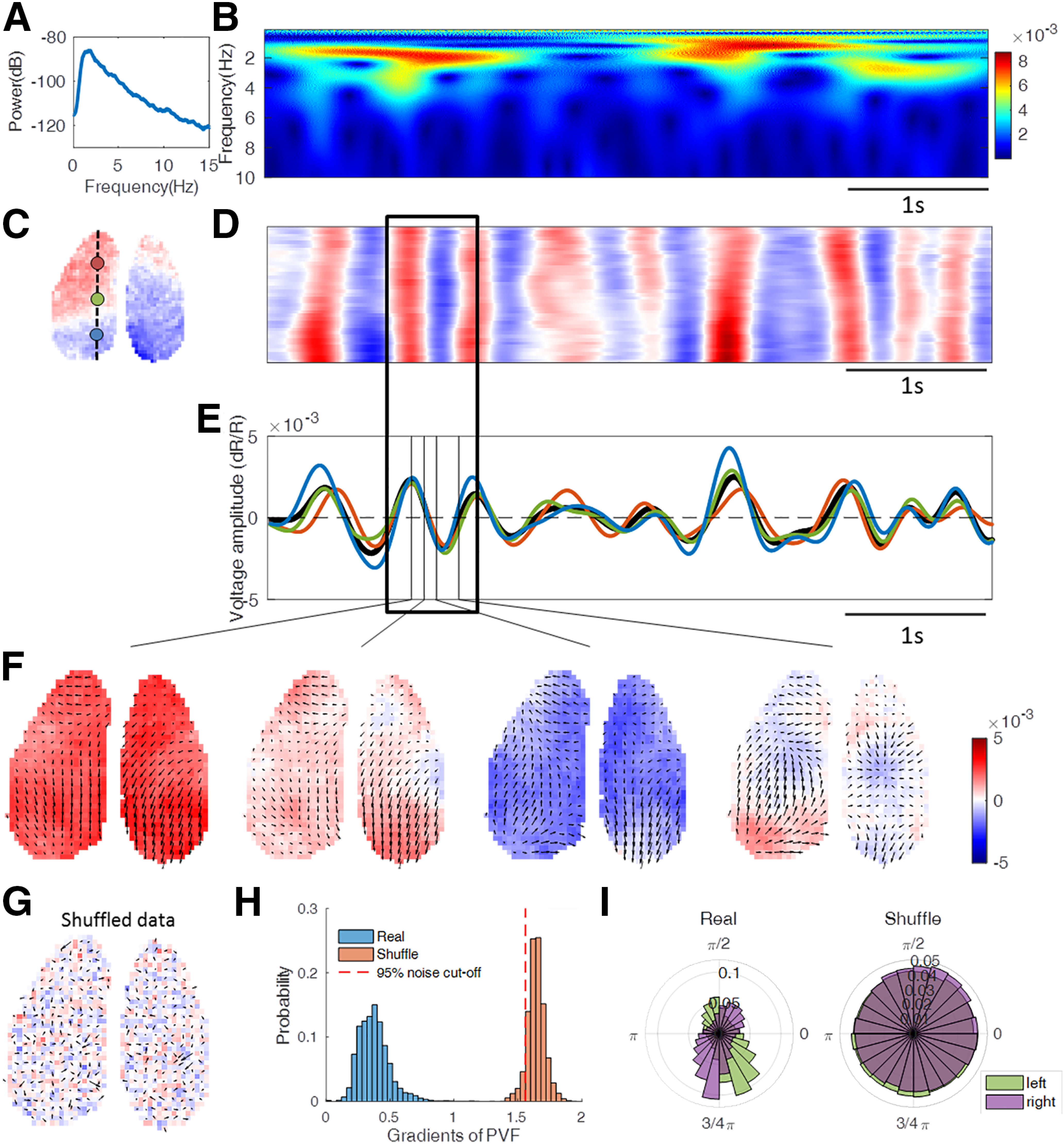
Spontaneous voltage activity as propagating waves characterized by the phase velocity field. ***A***, Power spectrum of spatially averaged voltage activity amplitude. ***B***, Time–frequency spectrum of voltage signals taken from the position marked by the blue spot in ***C***. Warmer color represents higher power. ***C***, Imaged cortical area for mouse 1. Background is the voltage map at the beginning of the time period shown in ***D***. ***D***, Example of GEVI-based voltage imaging data along a rostral–caudal line across the left hemisphere as indicated in ***C***; the colors represent the voltage amplitude (color bar is the same as in ***F***). ***E***, Voltage amplitude oscillation measured at the three spots; colors are as indicated in ***C***. The black line is the spatial averaged voltage amplitude. ***F***, Example of snapshots of phase velocity fields. The arrows are oriented in the wave-propagating direction, and their length indicates propagating speed. For clarity of visualization, only each second calculated vector is shown. The background colors represent the voltage amplitudes. ***G***, PVF of spatiotemporally shuffled data. Background colors are the same as in ***F***. ***H***, Probability distribution of gradients of PVFs obtained from real data and shuffled data. ***I***, Probability distribution of wave directions.

**Figure 2.**
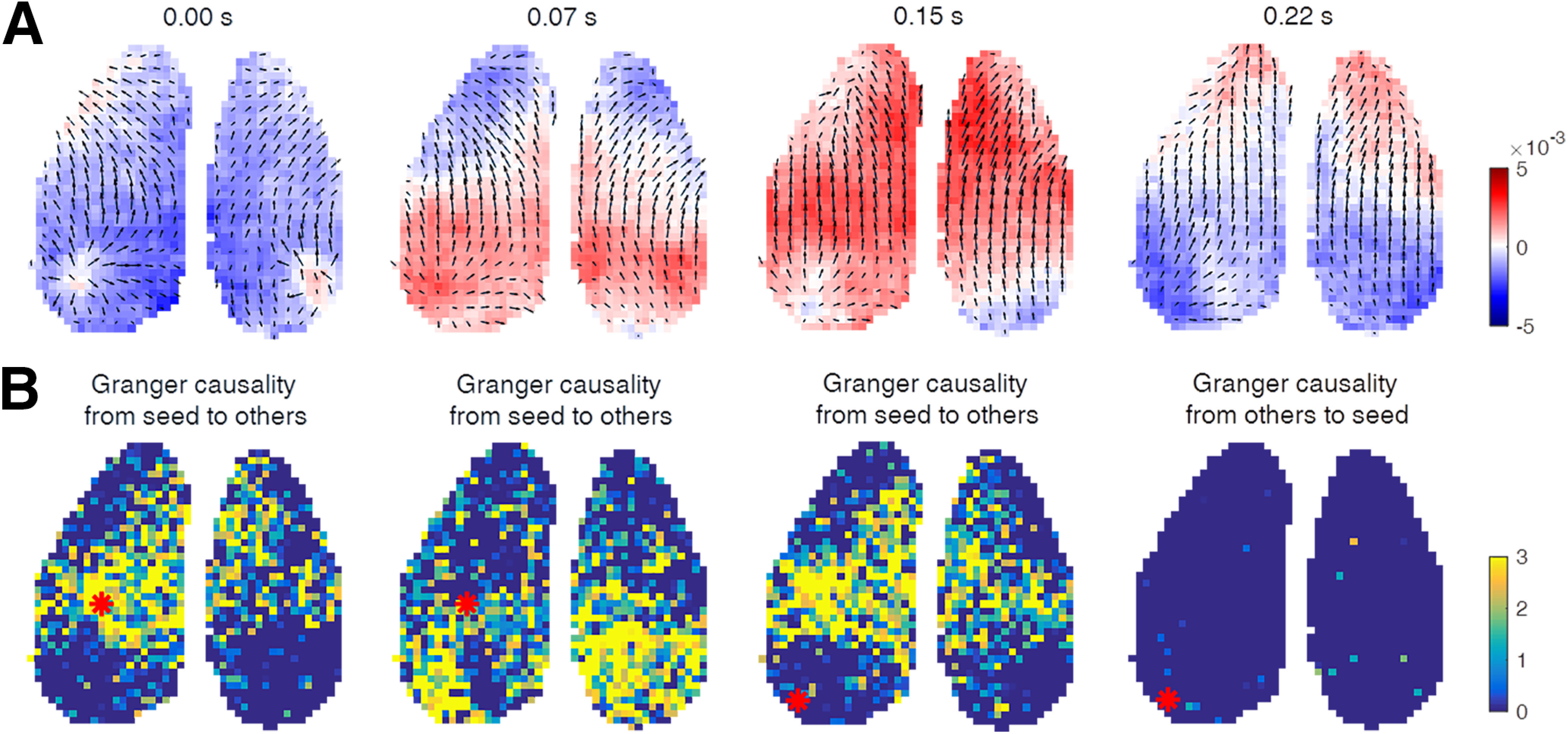
Granger causality of a traveling wave. ***A***, The phase velocity fields on a traveling wave propagate from posterior to anterior. The background color denotes the voltage amplitudes. ***B***, Granger causalities between a seed (red dot) and other pixels during period in ***A***. The background color denotes the value of *F*-Critical values in *F* statistic, for the 0.05 significance level, with *F* ≤ 0 referring to the absence of Granger causality, and *F* > 0 denoting the presence of Granger causality. Larger values can be considered as stronger causality. We set the maximum lag of the Granger analysis as five time steps.

We recorded voltage signals across the dorsal cerebral cortex using GEVI imaging in anesthetized mice. Consistent with previous studies, voltage power spectra indicate the presence of slow waves with a frequency of ∼2 Hz (Fig. 1*A*,*B*). For subsequent analysis, we bandpass filtered the signal from 0.5 to 4 Hz to increase the accuracy in the calculation of the phase velocity field (Fig. 1*F*). The spatiotemporal patterns of these spontaneous activities are well resolved when plotting the voltage signals from pixels residing along a rostral–caudal line in the imaged cortical space (Fig. 1*C*,*D*). These maps illustrate the occurrence of position-dependent time shifts, suggesting the existence of propagating waves. These plots alone may also indicate the presence of standing (nontraveling) waves (Volgushev et al., 2006), as suggested by the voltage signals measured at three positions on the line across the cortex (Fig. 1*E*). However, phase velocity field analysis reveals continuous and complex wave propagation (Fig. 1*F*), even in cases where the voltage signal (Fig. 1*D*) appears to be largely synchronized across distant locations. To validate our method of PVFs, we performed spatiotemporal shuffling (see Materials and Methods) on the raw voltage signals. Application of the identical data-processing pipeline resulted in unstructured PVFs (Fig. 1*G*). As the PVFs of shuffled data display less coherence in wave directions and speeds, gradients of PVFs (see Materials and Methods) were used to quantify the distinction of PVFs between real data and shuffled data (Fig. 1*H*). Smaller gradients indicate a larger coherence of local wave direction and speed. The clear separation of the distributions of gradients of PVFs from original and shuffled data demonstrates that the PVFs of unshuffled data do not emerge as a result of data processing but represent features of organized cortical activity. In the unshuffled data, the statistics of wave directions (Fig. 1*I*) showed that most of the waves propagate from rostral cortex to caudal cortex, while shuffled data do not exhibit a preference at all. In a previous analysis based on time delays in the positive peaks of LFP (Vyazovskiy et al., 2009), traveling waves appeared most frequently in anterior-to-posterior and posterior-to-anterior directions. Measurement by time delays in peaks is consistent with our analysis of phase velocity fields, but it only captures the dynamics at the peak of the oscillations. Phase velocity fields extend the characterization of wave propagation temporally to the whole time course of the activity wave and spatially to every imaged pixel and reveals far more complex propagation dynamics of waves during spontaneous activity (Movie 1). As the propagation path is consistent with the spatially organized sequential activation local circuits, wave propagations denote the information flow that could be validated by Granger causality (Granger, 1969; Seth, 2010). By calculating the pairwise Granger causalities, we found that neurons in the earlier part of a wave would have a causal role on neurons in its later part (Fig. 2). However, because these spontaneous wave patterns are ever changing, they may change directions in the next few time steps. This dynamical property makes the detection of a relatively stable causality between different areas unrealistic.

Movie 1. Complex wave dynamics during spontaneous activity. 10.1523/JNEUROSCI.0623-20.2021.video1

### Features of spontaneous large-scale activity waves

**Figure 3.**
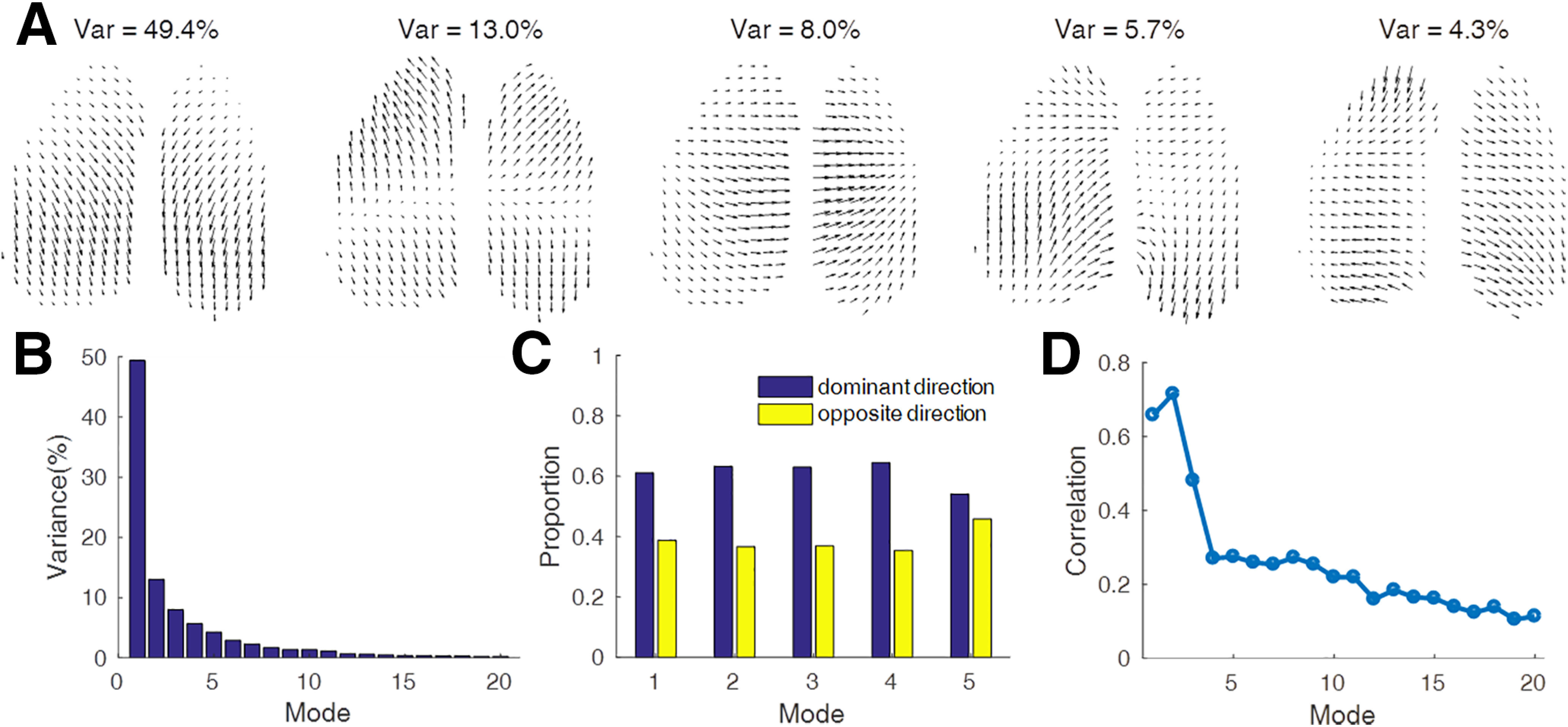
Principal modes of the phase velocity fields. ***A***, Top five modes calculated by singular value decomposition of all phase velocity fields from mouse 1 (each vector for each second pixel shown). ***B***, Variance distribution of the top 20 modes. ***C***, Proportion of the dominant and opposite directions of the top five modes. ***D***, Similarity of the top 20 aligned modes between different mice/trials (*N* = 13 trials, five mice).

Although the phase velocity fields displayed very rich and variable wave propagation dynamics, we wondered whether they exhibit features that are nonrandom, and can be consistently observed across many waves detected across animals. To address this question, we performed SVD on the PVFs (see Materials and Methods). The most frequent (principal) SVD modes reveal the typical wave propagation pathways, while the large number of small modes reflects the rich dynamics. The top five most dominant modes accounted for ∼80% of the total variance (Fig. 3*A*), and variance distribution of the modes decreased sharply (Fig. 3*B*). Each SVD mode represents patterns with a dominant direction (most frequently observed; illustrated in Fig. 3*A*) and the opposite direction (reversed for all pixels). The variance for each mode (both directions included) along with the relative proportion for each direction is indicated in Figure 3*C*. The first SVD mode accounts for nearly 50% of the variance and represents large-scale plane waves that are symmetrical across hemispheres. The less frequent mode 2 represents waves that, for their dominant direction, spread out from the somatosensory areas toward rostral and caudal cortices. The illustrated mode 3 waves spread from one hemisphere to the other hemisphere, while mode 4 waves have opposite directions of traveling waves in the two hemispheres. The illustrated mode 5 represents a sink (or source for opposite direction) in the somatosensory cortex on both hemispheres. Across different mice, modes 1 and 2 are consistently found to be the most frequent, but the frequency rank of the less frequent modes varies across animals. We used the modes shown in Figure 3*A* (mouse 1, trial 2) as a reference and aligned the top 20 modes in the other mice/trials according to their similarity to this reference, and the average of the absolute values of the Pearson correlation of reranked modes decreased with mode index (Fig. 3*D*). The high correlation coefficients of the top three modes indicate consistent features across animals and trials, and the sharp decrease in correlations across modes indicating increasing individual variability of the high-order modes.

### Classification and detection of wave patterns at smaller scales

**Figure 4.**
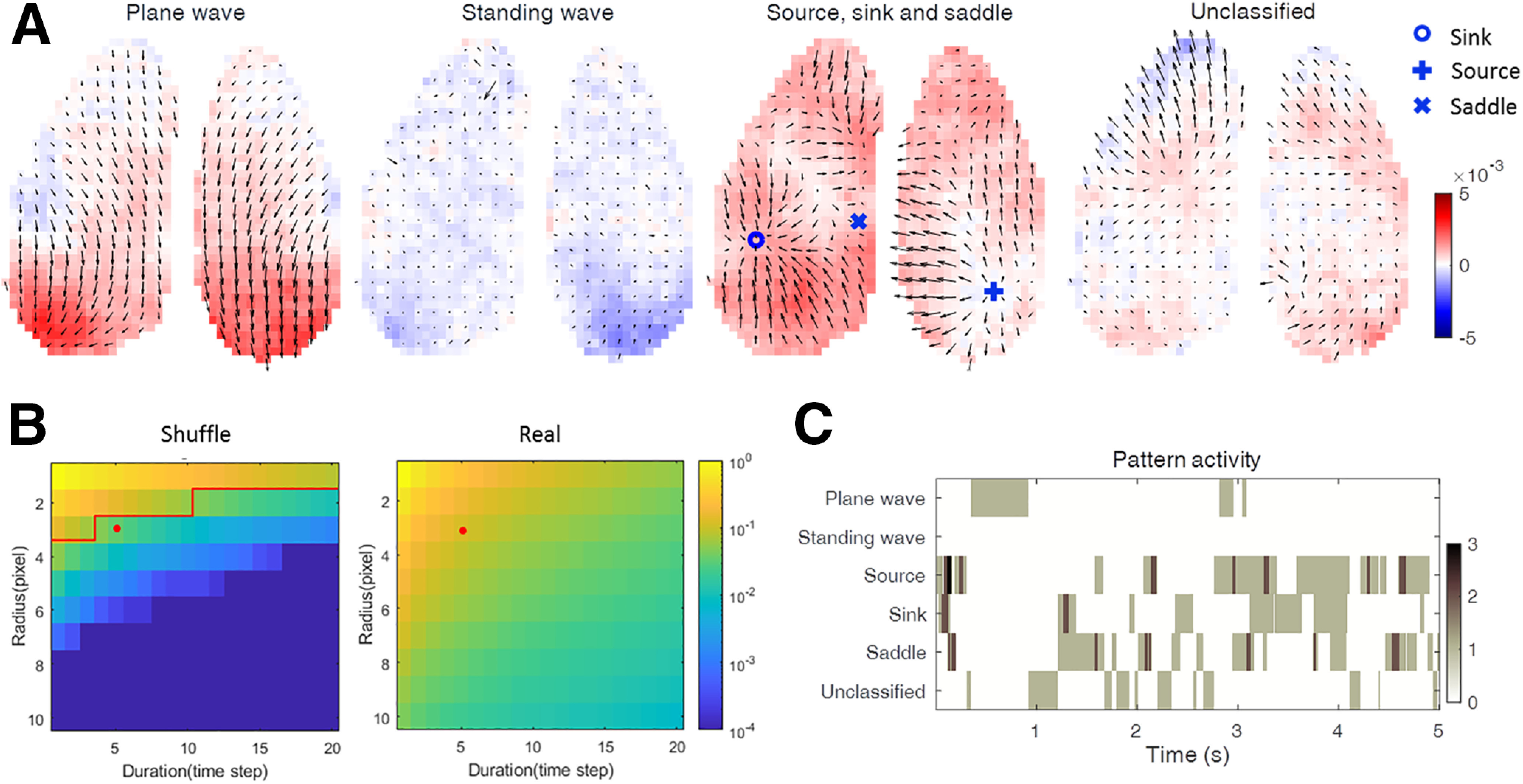
Classification and detection of specific wave patterns at the cortex-wide scale. ***A***, Examples of wave patterns: plane wave, standing wave, source, sink, and saddle. Vectors are shown for each pixel to facilitate the identification of individual patterns. ***B***, Probability of local wave pattern detection as a function of detection threshold [defined as a function of duration (*d*) and radius (*r*) threshold values]. Colors represent the probability that a local wave pattern is detected. Red line in shuffled data is the 95% confidence line for unshuffled data to be because of spatiotemporally organized activity. Red dot indicates the local wave detection threshold (5, 3) used in the following analysis. ***C***, Time course of the wave patterns from an example trial. Note that, when defined at the large scale, plane and standing waves occupy most of the imaged cortical space but are rare, whereas sources, sinks, and saddles may be locally restricted, but frequently occur. Color bar shows the number of waves of a certain type existing at time of a snapshot.

Using the SVD method, we found consistent propagation pathways for the cortex-wide activity waves. However, except for the top three SVD modes, the other modes are quite complex and capture less frequent events. A closer inspection of these SVD modes suggested that individual cortex-wide spatiotemporal wave patterns likely contain many smaller-scale features. To distinguish specific smaller-scale wave patterns, we defined the following five wave types (Fig. 4*A*; see Materials and Methods): plane wave (a traveling wave mainly in one direction); standing wave (a nonpropagating wave, effectively synchrony); source; sink; and saddle. If we did not detect any of these wave patterns in a given PVF frame, we counted such patterns as unclassified. Plane (traveling) and standing waves that occupy the whole imaged region occurred much less frequently than (smaller-scale) source, sink, and saddle waves (Fig. 4*C*). According to our classification criteria, waves analyzed across the whole imaged cortical area cannot be identified as plane wave and standing wave at the same time. However, plane waves and standing waves often occurred within smaller areas of the cortex and, when considering those smaller areas, can coexist with other wave patterns (sink, source, and saddle). For example, the unclassified pattern at the cortex-wide scale in Figure 4*A* contains a mixture of plane wave (rostral cortex) and standing wave (caudal cortex) in the left hemisphere. Thus, the classification of waves as plane or standing depended on the size of the cortical area analyzed. Because the source, sink, and saddle typically occupied small areas, we regard them as local wave patterns. Probability comparison of local wave patterns detected in real data and shuffled data (see Materials and Methods) indicates that large/long-lived local wave patterns are distinguishable from random nonorganized events. As shown in Figure 4*B*, with the increase of the detection threshold (defined by radius and duration of the detected event), the detection probability of localized waves decreased sharply when analyzing the shuffled data, while the probability in the real data decreased less steeply. A detection threshold of (5, 3) (Fig. 4*B*, red line) indicates 96.5% confidence that detected waves are not because of randomness that is also contained in the shuffled data. This detection threshold is used for the analysis presented in Figure 4 (and see Figs. 6, 7, 8, 9). Both wave pattern snapshots (Fig. 4*A*) and analysis of pattern activity occurrence in time (Fig. 4*C*), reveal that source, sink, and saddle wave patterns frequently coexist.

### Wave propagation across spatial scales

**Figure 5.**
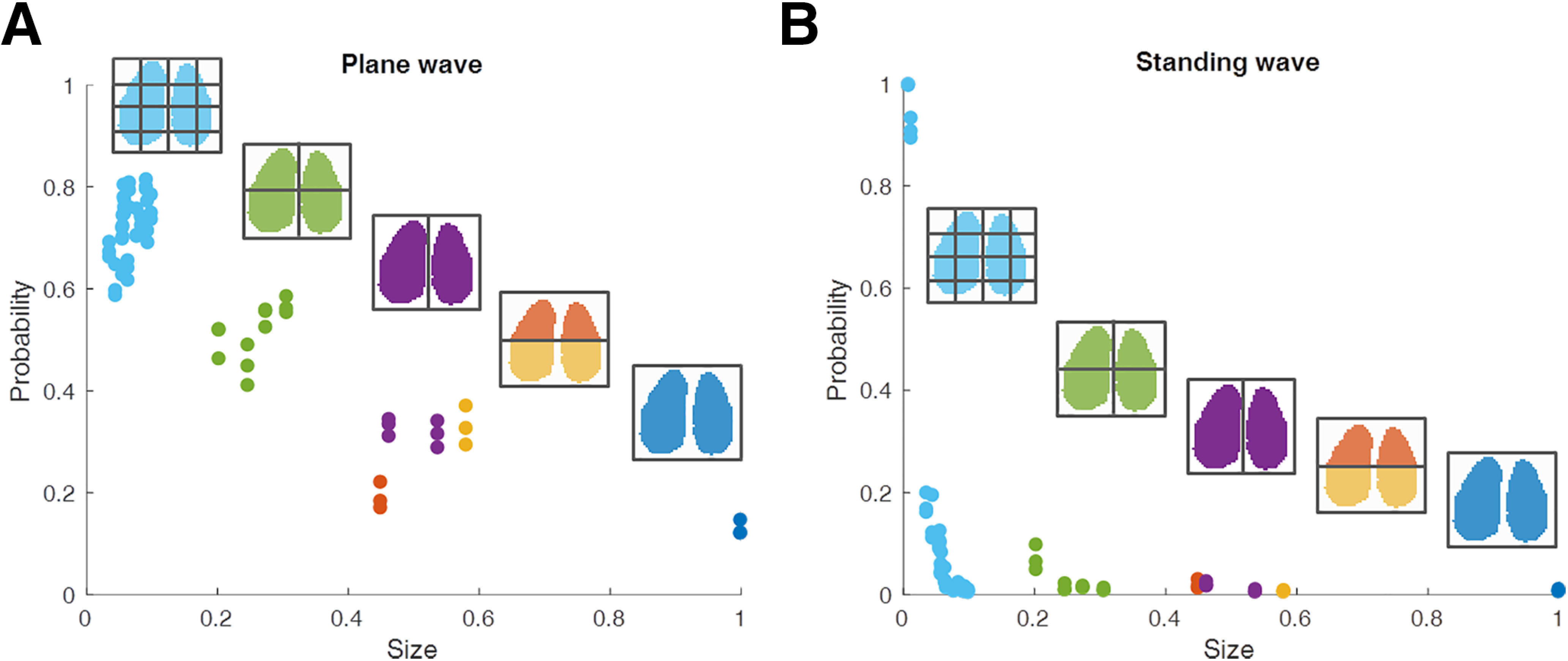
***A***, ***B***, Probabilities of detecting plane wave (***A***) and standing wave (***B***) decay with increasing ROI window size. The cortical image was divided into 16 parts (light blue), 4 parts (green), 2 parts (left and right, purple; rostral and caudal, orange and yellow), and 1 part (dark blue). The color of filled dots corresponds to coloration of the segments. The *x*-axis denotes the size of the cortical segments normalized to the total imaged area. Shown are the results of the three experimental trials from mouse 1. Similar results were obtained from the data of the other mice.

The high spatiotemporal resolution of our recordings allows us to test a key question, namely whether the plane waves and standing waves occur only at the scale of our whole imaging window, or also more locally. To address this question, we divided the imaged field of view into smaller spatial fields with 2, 4, or 16 regions of interest (ROIs; Fig. 5) and observed that the probability of detecting plane waves and standing waves sharply decreased with increasing size of the ROI. This indicates that large-scale waves represent only a small portion of detectable waves. Compared with plane waves, standing waves (including, according to our detection algorithm, also time points with no up-state-like activity in the detection window) are more sensitive to the detection window size (Fig. 5*B*), indicating that they very rarely occur globally. All these results are robust over the five mice analyzed. This observation underlines the notion that complex local waves contain rich information that may be missed if the recording resolutions are not high enough.

### Direction reversal of large-scale propagating waves by local wave patterns

**Figure 6.**
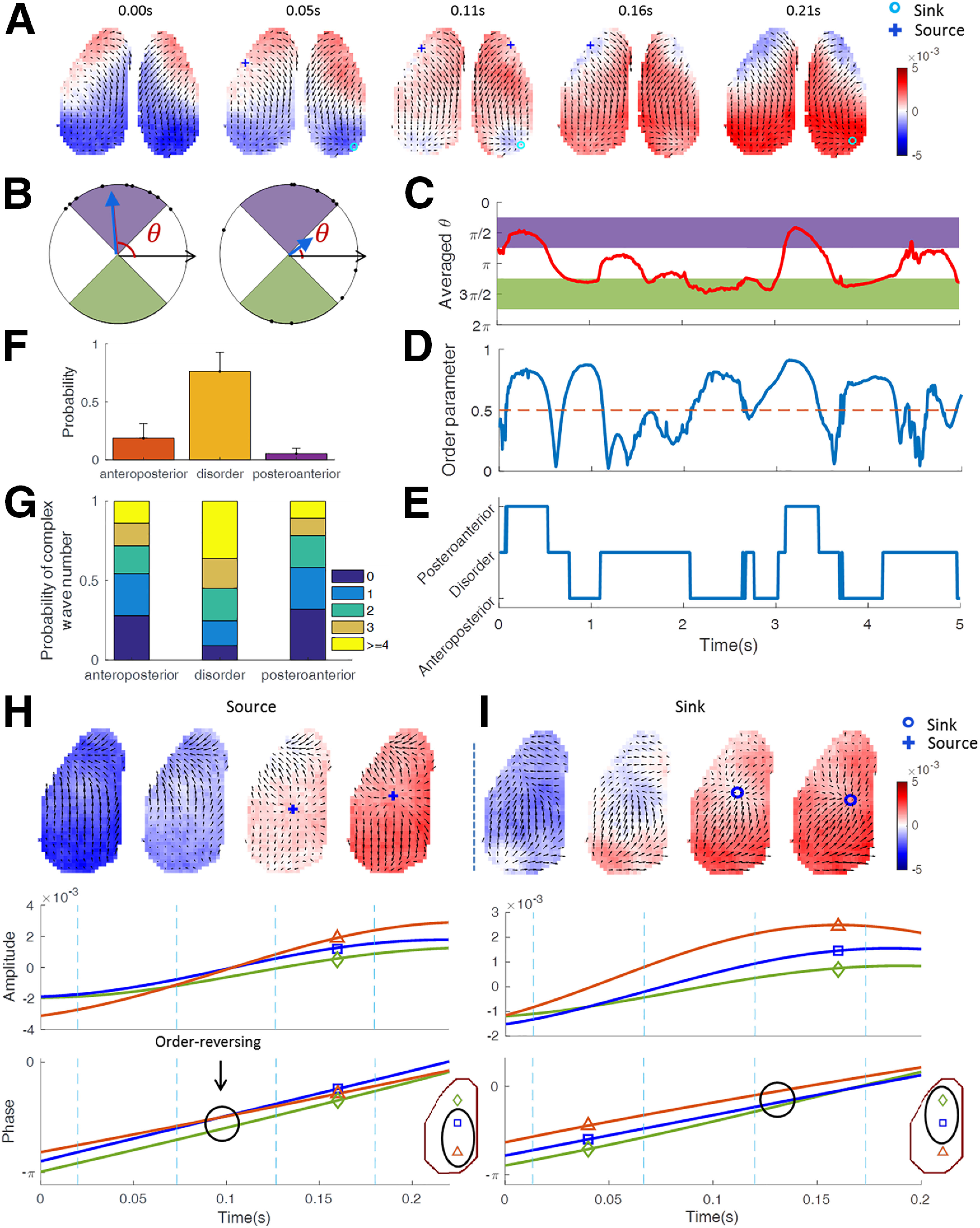
Reverse of propagation direction of traveling waves through interaction with local activity sources and sinks. ***A***, Anterior–posterior flow starts with anterior sources and ends with posterior sinks. ***B***, Schematic depiction of relative concordant directions (left) and disordered directions (right). Black dots on the circle represent local wave directions, the length of the blue vector is the order parameter and the angle of the vector is the average directionθ. ***C***, ***D***, The temporal evolution of $\theta and\overset{-}{v_{\varphi }}.$ We set π/4 < θ< 3π/4 as posteroanterior direction (purple) and 5π/4 < θ< 7π/4 (green) as the anteroposterior direction, and considered times of $\overset{-}{v_{\varphi }}$ < 0.5 as (large-scale) disordered. ***E***, The temporal evolution of wave propagation direction, according to the thresholding schemes in ***C*** and ***D***. ***F***, Probabilities of the anteroposterior, disorder, and posteroanterior states (*N* = 13 trials from five mice; mean ± SD). ***G***, Probabilities of occurrence of local complex waves during each large-scale state. The color code indicates the number of coexisting complex waves (total across sources, sinks, and saddles). ***H***, ***I***, Examples of the interaction of large-scale traveling waves and a local source (***H***) and sink (***I***). Top, Snapshots of the phase velocity fields (vectors for each second pixel are shown), at times indicated by vertical dashed lines in the middle and bottom panels. Color codes represent the instantaneous voltage amplitude. Middle, Instantaneous voltage amplitude (average of 3 × 3 pixels) recorded at the three ROIs are indicated on the inset. Bottom, Instantaneous phases corresponding to the 3 × 3 pixel-averaged signals for the three indicated ROIs. The reversal of phase orders is indicated by black circles.

Previous studies and our present data show that large-scale traveling waves preferentially propagate in an anteroposterior direction (Fig. 6*A*), but can occasionally reverse direction (Sanchez-Vives and McCormick, 2000; Massimini et al., 2004). However, the mechanism underlying this direction reversal is not known. We hypothesize that local complex waves may affect the direction of propagation of large-scale waves. As shown in Figure 5*A*, global plane waves rarely happened, let alone in the anteroposterior direction. Thus, we identified relatively large (but not necessarily whole field of view covering) traveling waves by setting an appropriate order parameter threshold (for details, see Materials and Methods). By lowering the order parameter to 0.5, we identified still relatively coherent, large-scale traveling waves, but allowing their temporal coexistence with other waves. As a measure of the instantaneous wave propagation direction, we calculated the spatially averaged propagation direction θ (Fig. 6*B*,*C*). This allowed us to identify waves propagating in the anteroposterior (5π/4<θ < 7π/4)or posteroanterior (π/4 < θ < 3π/4) directions. The direction measure θ together with the order parameter$\overset{-}{v_{\varphi }}>0.5$ identify relatively coherent traveling waves with either anteroposterior or posteroanterior directions. We classified other PVF patterns as disordered (Fig. 6*E*), but we shall note that disorder at the larger scale does not exclude order at a smaller scale. The time courses of θ and the order parameter $\overset{-}{v_{\varphi }}$(see Materials and Methods) showed interrelated fluctuations (Fig. 6*C*,*D*), as expected from a preferred propagation direction of coherent waves. Disorder at the large scale was found much more frequently than coherent PVFs, while in the coherent PVFs, the anteroposterior direction is more frequent than the reverse direction (Fig. 6*F*). These coherent waves in the anteroposterior or posteroanterior directions can coexist with local complex waves, while large-scale disordered PVF states can involve many local waves (Fig. 6*G*).

Next, we analyzed the formation of sources and sinks in the presence of coherent propagating waves. A source is defined as the origin of a wave with an oscillation phase that leads relative to the neighborhood. An example is shown in Figure 6*H*. Initially, oscillation phase over the visual cortex (Fig. 6*H*, orange triangle) is ahead, and the wave propagates from visual cortex to prefrontal cortex. At a later time, the visual cortex falls behind in phase along with a long period of increased activation, and a source emerges in the somatosensory cortex (Fig. 6*H*, blue square), accompanied by order reversing of the instantaneous phases between the ROI in the somatosensory cortex and ROI in the visual cortex. It appears that enhanced excitation in the somatosensory cortex accelerates the slope of the local depolarization (blue curve) and the emergence of a source in the PVF. The observed activity pattern also suggests that the prolongation of the activity in the visual cortex (Fig. 6*H*, middle, orange curve) is caused by excitatory inputs originating in the somatosensory source. These effects lead to phase order reversal and reversal of the propagation direction in visual cortex. As shown in Figure 6*I*, a large-scale wave initially propagated in the posteroanterior direction, but the activity wave in the somatosensory cortex (blue square) slowed down relative to the frontal cortical regions (green diamond), inducing phase order reversal and the changed wave propagating direction in the primary cortex from posteroanterior to anteroposterior.

**Figure 7.**
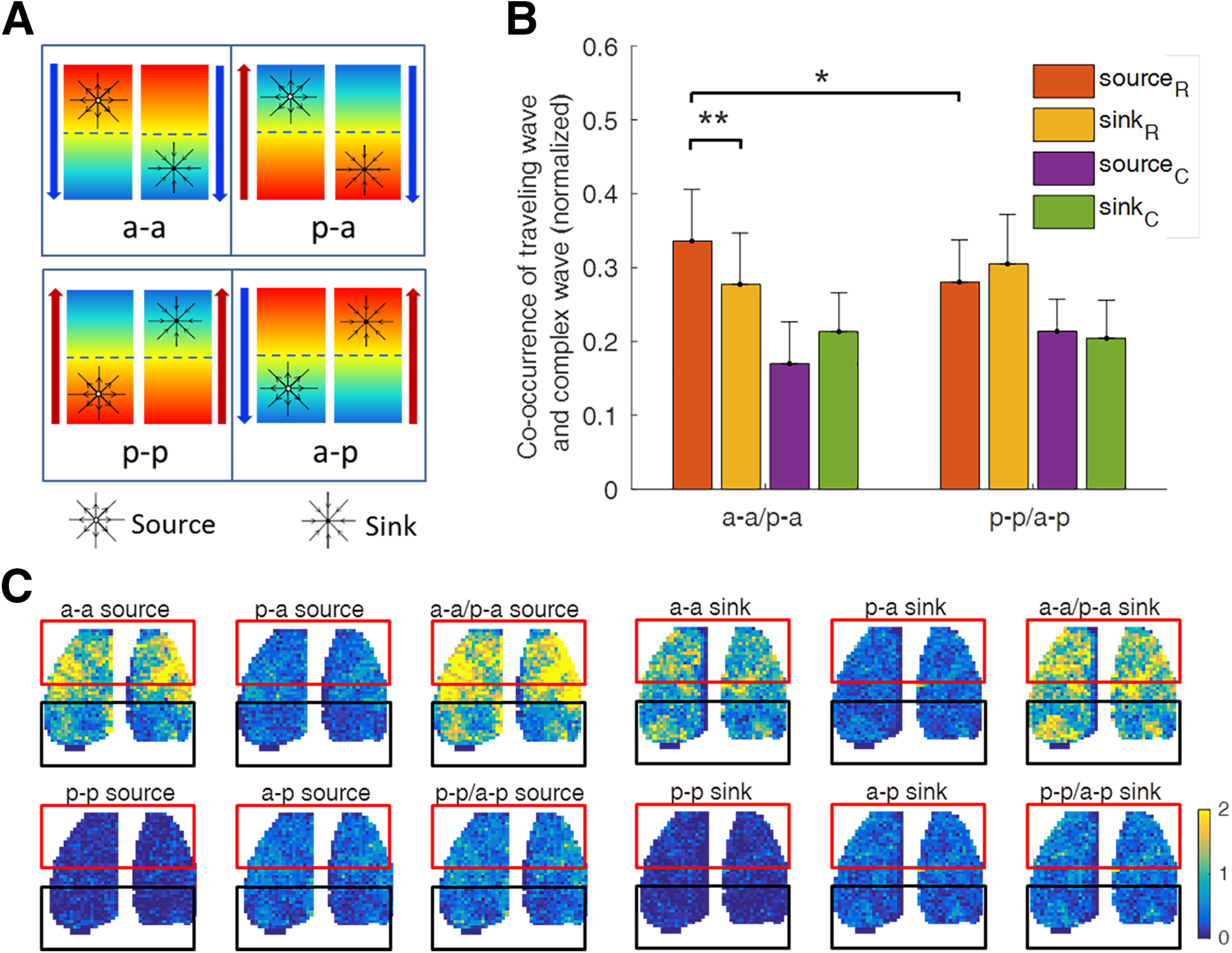
Interaction of plane wave and local waves induces transition of the wave-propagating directions. ***A***, Schematic illustration showing that the location and type of local waves can strongly determine the final wave propagation direction (Fig. 6*A*, example for the a-a condition). The four boxes represent different situations. The colored background indicates the initial plane wave-propagating direction (from red to blue), which is consistent with the arrow direction on the left. The arrow on the right side shows the final direction of the wave after the disordered period. The top (bottom) panel shows that the final direction tends to be anteroposterior (posteroanterior) regardless of the initial direction, if there is a rostral source (sink) and/or a caudal sink (source). ***B***, Probability of local wave patterns (sources and sinks in rostral and caudal cortex) during the large-scale disorder states on different conditions, grouped to compare the anteroposterior direction (a-a/p-a) or posteroanterior direction (p-p/a-p) in the final coherent waves following disordered interval. The legend “source_R_ (source_C_)” denotes a source located in rostral (caudal) cortex, and analogous for sink. The bars are the mean probability across five mice, and the error bar is the SD. The *p* values were calculated by Wilcoxon test: *p < 0.05, **p < 0.005. ***C***, Spatial distribution of the number of sources or sinks for different combinations of preceding and following large-scale wave directions (pooled data from five mice, 13 trails). The color shows the average number of the sources/sinks that occurred in the corresponding condition.

Having observed many cases like these two examples, we hypothesized that a sufficiently strong source and sink can change the direction of nearby ongoing traveling waves. As schematically depicted in Figure 7*A*, we more specifically hypothesized that waves of activity that propagate out from a source or toward a sink in the direction of a traveling wave will not affect the latter; otherwise, the direction of the traveling wave will be reversed through interaction with local sources and sinks (Fig. 6*H*,*I*).

To test this hypothetical scheme by statistical analysis, we considered the wave directions of two successive large-scale traveling waves interlaced by a large-scale disordered state. The directions of the large-scale wave can either remain the same [anteroposterior followed by anteroposterior (a-a) or posteroanterior followed by posteroanterior (p-p)] or can change [anteroposterior followed by posteroanterior (a-p) or posteroanterior followed by anteroposterior (p-a)]. We counted the occurrence of sources and sinks that occurred during the last quarter interval of the previous disordered state or during the first quarter interval of the following large-scale wave for each of the conditions a-a, a-p, p-a, and p-p. Based on our hypothesis that the direction of the large-scale traveling wave depends on the nature and localization of preceding local waves, we combined a-a/p-a as one scenario and p-p/a-p as another scenario. Because it is unlikely that sources or sinks localized in the center of the cortex would influence the direction of the large-scale waves emerging in the posterior or anterior portion of the cortex, we only counted the sources and sinks in the anterior area (Fig. 7*C*, red box) and posterior area (Fig. 7*C*, black box) of the cortex. As shown in Figure 7*C*, we plotted the average spatial distribution of sources and sinks for each of the scenarios (five mice, 13 experimental trials). For statistical analysis, we first normalized the small wave counts for the a-a/p-a scenario and the p-p/a-p scenario by the total number of anteroposterior waves and posteroanterior waves (Fig. 6*F*). We also normalized the number of sources and sinks because sources were more frequently detected than sinks (Fig. 7*B*). When examining the scenario a-a/p-a, we noted that in the rostral cortex there is a much higher probability of sources than sinks, while in the caudal cortex there is a higher probability of sinks than sources. Furthermore, rostral sources were found more frequently in the scenario a-a/p-a than in the scenario p-p/a-p (Fig. 7*B*). Consistent with our hypothesis, these results suggest that complex waves during initialization and early progression of the large traveling waves can influence the direction of the latter.

### Interaction of local wave patterns

**Figure 8.**
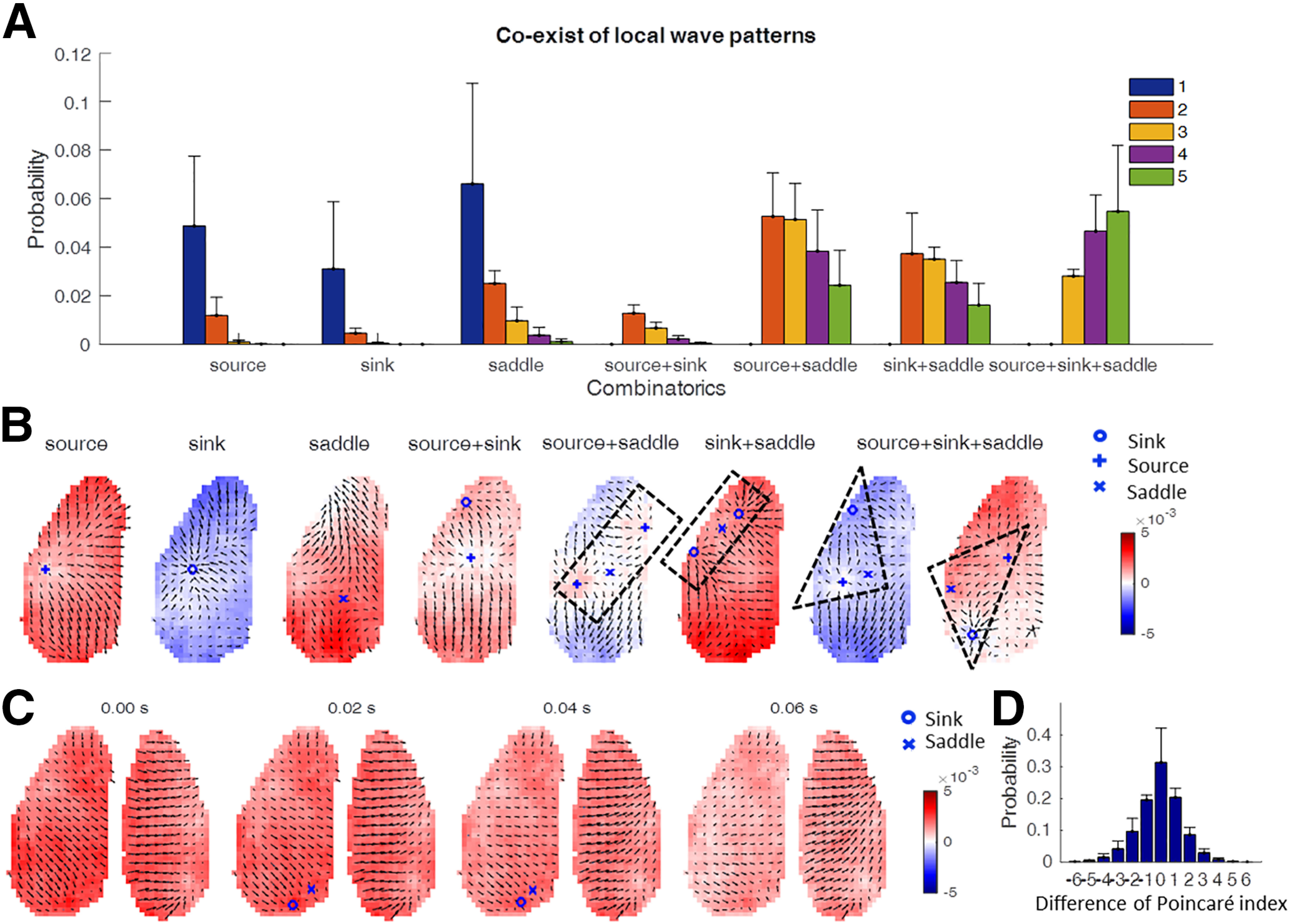
Interaction of waves. ***A***, Coexistence of local wave patterns. We considered the following seven combinatory situations: only source; only sink; only saddle; source and sink; source and saddle; sink and saddle; source, sink, and saddle. Shown are the probabilities of occurrence/co-occurrence in field of view with the number of detected waves indicated by the color code (*N* = 13 trials from five mice, mean ± SD). ***B***, Top row, Examples of phase velocity fields for every situation counted in ***A***. ***C***, Phase velocity fields with paired appearance and disappearance of local wave patterns with Poincaré index (+1 for source or sink, −1 for saddle). Background colors denote the voltage amplitude. ***D***, The probability of changes of total Poincaré index between successive time steps.

Most PVF snapshots show only a single source, sink, or saddle (Fig. 8*A*). Among all occasions where two local complex waves coexisted, the most frequent combination was source–saddle pairs. Coincidence of three or more local wave patterns was also observed. To gain further insights into the spatial characteristics of the interaction of local waves, we analyzed the representative phase velocity fields for each combination of coinciding local wave patterns (Fig. 8*B*). This analysis revealed that the saddle pattern typically emerged from the interaction of two complex waves regardless of wave type (source or sink). Moreover, two sources or two sinks would produce a saddle between them (Fig. 8*B*, highlighted by black dot rectangle), while one source and one sink would produce a saddle in a location away from the line segment and form a triangle (Fig. 8*B*, highlighted by the black dotted triangles).

Is there an organization principle that governs the interactions of local wave patterns? According to the Poincaré–Hopf theorem of vector flow fields (Hopf, 1927), if the vectors along the boundary do not change in topology, the emergence of local wave patterns within the boundary pairwise maintain the total Poincaré index (+1 for source or sink, −1 for saddle). An example of the emergence and disappearance of a sink–saddle pair is shown in Figure 8*C*. We used the difference of the Poincaré index at time *t* + 1 and *t* to monitor the (balanced) emergence of complex waves. The statistical analysis of data from 13 180 s recording sessions across five mice confirmed that the total Poincaré index remains mostly unchanged (Fig. 8*D*). This in turn confirms that the appearance or disappearance of sources or sinks is accompanied by the emergence of a saddle. The events with nonzero changes of total Poincaré index are still consistent with the Poincaré–Hopf theorem since the topology of the vectors along the boundary change when the complex waves move across the boundary.

### Relating preferred locations of sources, sinks, and saddles to cortical hierarchy

**Figure 9.**
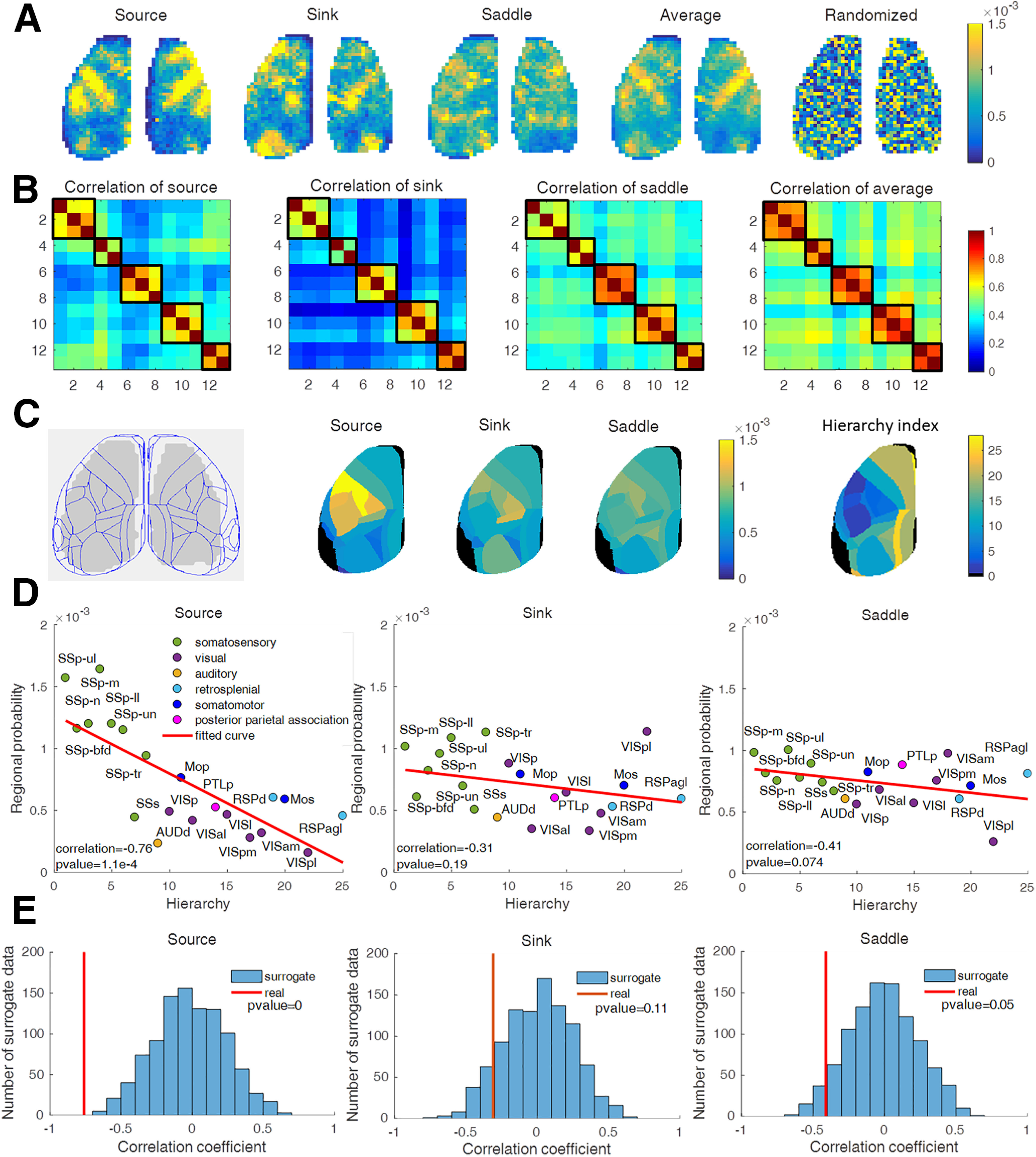
Spatial distributions of singularities and their relationship with cortical structural hierarchy. ***A***, Pixel-wise distribution of the average occurrence probability for each wave pattern type (source, sink, and saddle), total complex waves (average), and one example of the shuffled data from the source pattern (randomized). *N* = 13 trials across five mice. ***B***, Correlation coefficients of the spatial distribution of local wave patterns. The *x*-axis and *y*-axis indicate the experiment index: mouse 1, 1–3; mouse 2, 4–5; mouse 3, 6–8; mouse 4, 9–11; mouse 5, 12–13. Black box shows the correlation of trails within the same mouse. ***C***, Left, Voltage-imaging data after functional registration into the Allen Mouse Brain Atlas. Middle, Cortical region maps (left hemisphere) of cortex-wide average probability (pooling across the two hemispheres) of source, sink, and saddle wave patterns. Right, Cortical region maps (left hemisphere) of hierarchy index derived from the study by Fulcher et al. (2019). Regions with black colors are not covered by the field of view of the voltage-imaging datasets used. ***D***, Scatter plot of the correlation between regional source/sink/saddle probability and the hierarchy index of the corresponding cortical regions. ***E***, Histogram distributions of the correlation coefficients between the hierarchy index and spatially randomized singularity data.

Finally, we investigated whether there are preferred locations for sources, sinks, and saddles, and, if so, whether they relate to structural features of the mouse brain. From the maps of cumulative localization for each wave pattern type (Fig. 9*A*; *N* = 13 trials from five mice), the probabilities of source, sink, and saddle occurrences all show uneven spatial distribution that are similar across individual mice (Fig. 9*B*). The similarity between different datasets from a given mouse is higher than the similarity among different mice (Fig. 9*B*), conferring some degree of individuality. Despite this, the high similarity (Fig. 9*B*) of spatial distribution of singularity (wave center) across mice suggests that the preferential spatial locations of the source, sink, and saddle patterns may be related to the underlying anatomic architecture of the brain. After registration of the wave localization maps onto the Allen Mouse Brain Atlas, we generated the averaged the probability values among pixels in each brain area (Fig. 9*C*). When comparing these wave pattern probability maps against the common hierarchical gradient (as a choice of structural measure; Fulcher et al., 2019), we observed significant negative Pearson linear correlations between the regional probabilities of sources (correlation = −0.76, *p* = 1.1e-4) and the hierarchical index across the cortical areas, while sinks (correlation = −0.31, *p* = 0.19) and saddles (correlation = −0.41, *p* = 0.074) have some trends without significance (Fig. 9*D*). No dependencies were observed in the spatially randomized singularity distribution (Fig. 9*E*).

## Discussion

By taking advantage of high-resolution voltage imaging of the mouse cortex, here our work identifies key spatiotemporal organization properties of spontaneous activity at the cortex-wide level. These properties include the following. (1) Waves of activity propagate along many different pathways but a few dominant principal velocity field modes account for a large proportion of variance (Figs. 1, 3). (2) Global plane waves and standing waves are rare (Fig. 4). (3) Different complex wave patterns can occur either in isolation or in combination, as expected from the Poincaré–Hopf theorem. The direction of waves can be reversed as a consequence of their interactions (Figs. 6, 7), and interactions of two local waves, no matter whether source or sink, generate a saddle pattern (Fig. 8). (4) The spatial distributions of source, sink, and saddle are similar across individual mice, and the incidence of local wave patterns correlates with features of the underlying cortical hierarchical architecture, as characterized by the covariation of diverse measurements including gene expression, intracortical axonal connectivity, the ratio of T1-weighted to T2-weighted (T1w:T2w ratio) (myelination contents), and interneuron cell density (Fulcher et al., 2019). In particular, the spatial distribution of sources is highly related to the hierarchical gradients derived from the structural variation (Fig. 9).

The SVD shows that the top two principal modes reflecting two typical wave-propagating pathways are highly conserved across experimental trials and animals (Fig. 3). The first dominant principal SVD modes represent cortex-wide traveling waves that propagate preferentially along an anterior–posterior (with a smaller medial–lateral component) axis with hemispheric symmetry. This result is consistent with intracellular and extracellular recordings that revealed that spontaneous slow waves under anesthesia preferentially propagate in the anterior–posterior direction and sometimes propagate reversely (Sanchez-Vives and McCormick, 2000). The delta band calcium signals under anesthesia also provided evidence of traveling waves in directions along the anterior–posterior axis of the mouse cortex (Mitra et al., 2018). Corresponding large-scale propagation directions were shown using high-density EEG recordings in humans (Murphy et al., 2009), in cats (Volgushev et al., 2006), and, in part, in rodents (Vyazovskiy et al., 2009).

The second principal mode appears as a source localized medially halfway along the rostral–caudal axis. The third principal mode represents sweeps of activity from one hemisphere to the other. This contrasts to the much more frequent homeotopic patterns and may relate to left–right asymmetry (lateralization).

The wave propagation represents sequential activation of neural circuits along the propagation pathway; thus, it generally denotes the information flow in the brain. In the special case of anesthesia, the orderly propagation of neuronal activity along typical pathways gives rise to sequence-like activity, which gives rises to causality in the dynamics. Similar dynamical patterns during slow-wave sleep may interplay with spike timing-dependent synaptic plasticity required for memory consolidations or synaptic downscaling (Tononi and Cirelli, 2003). At least some of these processes are likely to occur during slow-wave sleep. Moreover, spontaneous traveling cortical waves in the local cortical region have been reported to gate perception in behaving primates (Davis et al., 2020) and to modulate neural excitability by locking to spikes (Townsend et al., 2015). It is therefore plausible to consider that cortex-wide waves would modulate large-scale propagation of stimulus-induced neuronal activities, thus coordinating activities at different cortical areas. Therefore, an important issue to be addressed in future studies is to which extent these specific wave directions/trajectories determine activities observed in awake states.

We noted that the spontaneous activities propagate across cortical space without being affected by the boundary of anatomically and functionally defined cortical areas while sensory stimuli-driven activities, at least initially, are (Petersen et al., 2003; Xu et al., 2007; Mohajerani et al., 2013; Song et al., 2018). Stimuli-driven activities may be confined to one brain region, for instance in sensory representations that trigger no behavioral response. However, the predictive coding theory and simple stimulus response models would require the involvement and coordination of distributed brain systems (Arieli et al., 1996). In both frameworks, spontaneous activities could serve as a cortex-wide coordinating mechanism for performing distributed dynamical computation (Gong and van Leeuwen, 2009).

We observed that different types of local waves can exist alone (Fig. 6) or in combination (Fig. 8) at any location of the imaged cortex. Saddles typically emerge from the interaction of two local waves, similar to observations in the middle temporal area of marmoset cortex (Townsend et al., 2015), and such interactions might endow neural circuits with the computational capacity of integrating distributed events happening at different space and time. We also indicated that the emergence and disappearance of local wave patterns are in agreement with predictions of the Poincaré–Hopf theorem. The observed small fluctuations in the total Poincaré index may be explained by several factors including the fact that (1) the imaged portion of the mouse cortex is not perfectly flat, which complicates geometrical analysis, and that (2) our spatiotemporal resolution and the signal-to-noise ratio may not be high enough to resolve all the local wave patterns.

At present, it is unclear whether global and local waves are mechanistically distinct. However, their interactions described here suggest that they, at least, share or compete for the same cellular mechanisms. Indeed, large-scale waves propagate out from sources and into sinks; a rostral source or a caudal sink or their combination favors wave propagation in the anteroposterior direction (Fig. 7). If the local waves are of low amplitude (i.e., recruiting only a small fraction of local neurons), larger global waves may be simply sweeping through. Otherwise, if a local complex wave is of larger amplitude (i.e., recruiting a larger fraction of neurons), the complex wave will expand and eventually dominate a larger fraction of the cortical space. Meanwhile, global plane waves may also propagate from sources to sinks with the sources and sinks outside our optical imaging window. All this evidence supports the idea that local and brain-wide waves share the same cellular and synaptic mechanisms.

The organization properties of brain waves is a long-standing interest in neuroscience (Muller et al., 2018), and recent studies have begun extending the classical description of brain waves in terms of temporal synchrony (Nir et al., 2011) to the spatial and temporal domains (Takagaki et al., 2011; Zhang et al., 2018; Davis et al., 2020). The advance in our current work is the application of the analysis on data of high spatial and temporal resolution as well as of large coverage (the major part of dorsal cortical hemispheres), revealing a variety of wave organization properties in a quantitative way. In this study, we focused on the anesthetized state, and we expect that during the fully awake state, the cortex-wide dynamics could exhibit more localized wave patterns with complex interactions; this prediction can be tested in the future. In this study, we mainly investigated the wave patterns of slow oscillations. It has been widely observed that the phases of low-frequency oscillations are coupled to the amplitudes of high-frequency oscillations in the brain (Canolty and Knight, 2010). Therefore, it would be interesting to investigate how this cross-frequency coupling influences or is influenced by the underlying wave patterns (Townsend and Gong, 2018). By revealing the organization properties of propagating waves at the cortex-wide level, the present study lays a ground for further exploring these key questions in future studies.
